# Revisiting the impact of age and molecular subtype on overall survival after radiotherapy in breast cancer patients

**DOI:** 10.1038/s41598-017-12949-5

**Published:** 2017-10-03

**Authors:** Jian-Hua Mao, Paul J. van Diest, Jesus Perez-Losada, Antoine M. Snijders

**Affiliations:** 1 Biological Systems and Engineering Division, Lawrence Berkeley National Laboratory, Berkeley, California, USA; 2 Department of Pathology, University Medical Center Utrecht, Utrecht, The Netherlands; 3 Instituto de Biología Molecular y Celular del Cáncer (CIC-IBMCC), Universidad de Salamanca/CSIC, Salamanca, 37007 Spain; 4 Instituto de Investigación Biomédica de Salamanca (IBSAL), Salamanca, 37007 Spain

## Abstract

Adjuvant radiotherapy (RT) in breast cancer (BC) is often used to eradicate remaining tumor cells following surgery with the goal of maximizing local control and increasing overall survival. The current study investigated the impact of age and BC molecular subtype on overall survival after RT using a meta-analysis of the METABRIC and TCGA BC patient cohorts. We found that RT significantly prolonged survival across the whole BC patient population. The survival benefit of RT was predominantly observed in stage II BC patients treated with breast conserving surgery. Patients were then stratified by age and molecular subtype to investigate survival rate associated with RT. An increase in survival for the luminal-A and basal BC molecular subtypes was observed after RT. Stratifying patients based on age revealed that increased survival was restricted to younger patients (≤60 years of age at diagnosis). There was a significant survival benefit of radiotherapy for younger patients with tumors of the luminal A and basal molecular subtypes. Patients with other breast tumor subtypes or older breast cancer patients did not seem to benefit effects of RT. Therefore, alternate local treatment strategies should be considered for older, luminal B, and HER2 driven BC patients.

## Introduction

Breast cancer (BC) is the leading female malignancy and the second leading cause of cancer deaths in U.S. women, with tumor metastasis being the underlying cause in most of these breast cancer related death^1,2^. Breast carcinogenesis is a multi-step process in which epithelial cells accumulate genetic alterations, which in a permissive tissue microenvironment progress towards malignancy and may then metastasize to distant organs. Gene expression profiling has been used to classify breast cancers into different molecular subtypes^3–6^. Advances in imaging technologies, screening programs and heightened public awareness of breast cancer have resulted in an increase in the diagnosis of early-stage breast cancer^7–9^. Furthermore, adjuvant therapy has reduced the risk of recurrence and improved overall survival from BC^10^. Radiotherapy is a well-established adjuvant treatment modality following breast cancer surgery. However, not all patients who receive radiotherapy benefit from it and could have been spared the treatment-associated side-effects including short-term effects such as skin erythema and fatigue and later side effects including telangiectasia, impaired cosmesis and angiosarcoma^11–13^. Separating patients who benefit from those who do not benefit from radiotherapy remains however challenging, and current clinical practice therefore considers radiotherapy for all patients undergoing breast cancer surgery. The availability of large cancer genomic data sets in combination with clinical data allows for exploring unbiased approaches to identify patients that do and do not benefit from radiotherapy and biomarkers that can predict the response to radiotherapy. Here, we combined two large breast cancer databases to address the impact of age at diagnosis and breast cancer molecular subtype on response to radiotherapy.

## Results

### Radiotherapy improves overall survival in breast cancer patients

Solid evidence has shown that radiotherapy after BC surgery leads to increased patient survival. To further validate this observation, we used two large BC patient cohorts (METABRIC and TCGA), which contain clinical data including radiotherapy, molecular subtype, age, overall survival (OS) and other patient characteristics. We first investigated the demographic differences between patients that received radiotherapy versus those that did not using the METABRIC and TCGA data (Table 1). A higher proportion of young patients, patients receiving breast conserving therapy and patients with high grade and late stage tumors received radiotherapy (Table 1). Overall we found that patients who receive radiotherapy survive significantly longer compared to those who did not receive radiotherapy in both datasets (Figure S1; METABRIC: p = 0.007; TCGA: p = 1.12E-04).

**Table 1:** Distribution of clinical characteristics of METABRIC and TCGA breast cancer cohorts.

	METABRIC			TCGA		
	Radiotherapy		p-value	Radiotherapy		p-value
	No	yes		No	yes	
Age (mean + /− st.dev)	63.1 (12.8)	59.7 (12.9)	1.05E-08	59.8 (14.1)	56.6 (12.0)	1.69E-04
Age ≤ 60 years*	303	572		223	322	
Age > 60 years*	500	599		206	220	
Tumor size (mm; mean + /− st.dev)	25.8 (13.3)	26.5 (16.7)	0.098	Na	Na	Na
Surgery*			4.31E-14			2.27E-14
Breast conserving	80	705		48	231	
Mastectomy	712	458		269	200	
ER status*			0.29			0.81
neg	158	316		91	112	
pos	649	857		315	405	
PR status*			0.015			0.89
neg	359	581		130	164	
pos	448	592		273	353	
Histological Grade*			1.42E-09	Na	Na	Na
I	85	84				
II	353	418				
III	309	643				
Stage*			4.91E-09			7.15E-07
0	9	3				
1	212	289		69	85	
2	265	560		279	282	
3	18	100		62	158	
4	5	5		10	7	
PAM50 subtype*			6.91E-05			5.97E-01
Luminal A	322	378		159	188	
Luminal B	176	299		66	96	
Her2	90	134		24	25	
Basal	68	141		46	69	

*Number of patients.

### Effect of radiotherapy on patient survival is dependent on clinical stage and surgery

**Figure 1:**
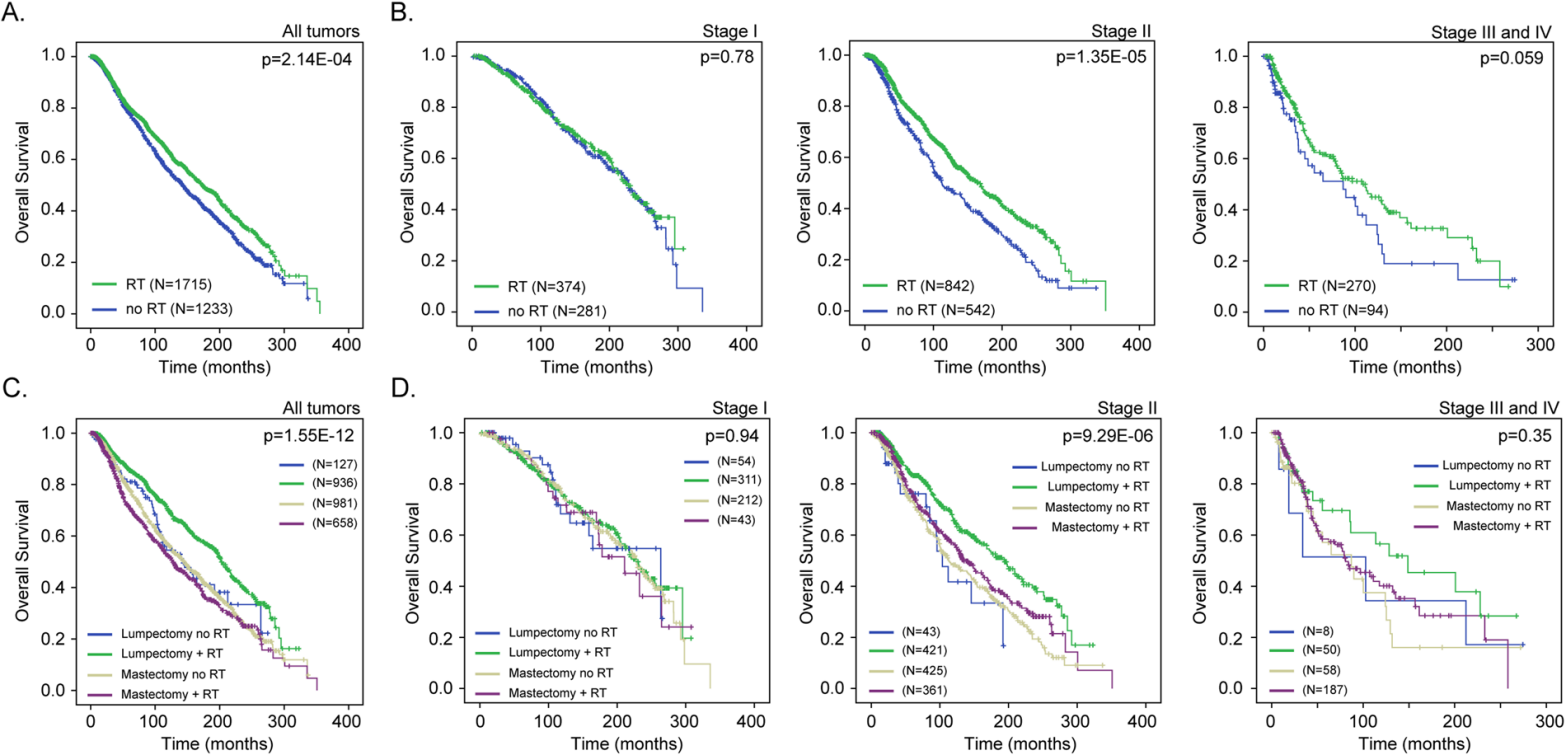
Clinical stage and surgery significantly influence survival outcome after radiotherapy. (**A**) Kaplan-Meier overall survival meta-analysis of radiotherapy (RT) benefit in the combined METABRIC and TCGA cohort (N = 2948; p = 2.14E-04). (**B**) Influence of clinical stage on RT survival benefit. (**C**) Influence of surgery type (lumpectomy vs mastectomy) on RT survival benefit. (**D**) Combined effect of clinical stage and surgery type on RT survival benefit.

To study how clinical stage and surgery influence the effect of RT on patient survival, we first combined the METABRIC and TCGA cohorts and confirmed that radiotherapy significantly increased patient survival in the combined patient cohort (Fig. 1A; p = 2.14E-04). We then stratified the patient cohort by clinical stage and assessed whether RT offered a survival benefit in different subgroups. We found that radiotherapy increased patient survival in patients with stage II disease (Fig. 1B; p = 1.35E-05), whereas a tendency for survival benefit was observed in patients with stage III/IV disease (Fig. 1B; p = 0.059). No benefit was observed in patients with stage I disease (Fig. 1B; p = 0.78). We further stratified the patient cohort by surgery type (lumpectomy *vs*. mastectomy). Our data showed that in the cohort of patient that underwent lumpectomy, RT significantly increased survival (Fig. 1C; p = 0.012), especially in patients with stage II disease (Figure 1D; 0.014). In the cohort of patients that underwent mastectomy, RT had a tendency to increase survival in stage II patients (Fig. 1C; p = 0.07).

### Impact of radiotherapy on patient survival is independent of clinical factors

To determine if the impact of radiotherapy on patient survival was independent of age at diagnosis, tumor size, estrogen-and progesterone-receptor status, tumor grade and molecular subtype (as determined by Pam50) we used multivariate Cox regression with these factors including radiotherapy as covariates. In multivariate analysis the difference of OS attributable to radiotherapy remained significant (HR = 0.873: 95% CI: 0.771-0.988; p = 3.16E-02). We also found that age, tumor size, ER status, tumor grade and PAM50 subtype were significantly associated with OS (Table 2).

**Table 2:** Prognosis factors in multivariate analyses.

Factors	p-value	Hazard Ratio (HR)	95% CI for HR	
			Lower	Upper
Radiotherapy	3.16E-02	0.873	0.771	0.988
Age (years)	5.02E-37	1.036	1.031	1.042
Tumor size (mm)	7.44E-19	1.014	1.011	1.017
ER status	6.90E-04	0.667	0.528	0.843
PR status	1.65E-01	0.900	0.777	1.044
Grade	2.29E-03			
Grade II vs I	1.52E-01	1.200	0.935	1.539
Grade III vs I	3.74E-03	1.462	1.131	1.891
Pam50 subtype	1.92E-04			

### Molecular subtype specific impact of radiotherapy on patient survival

**Figure 2:**
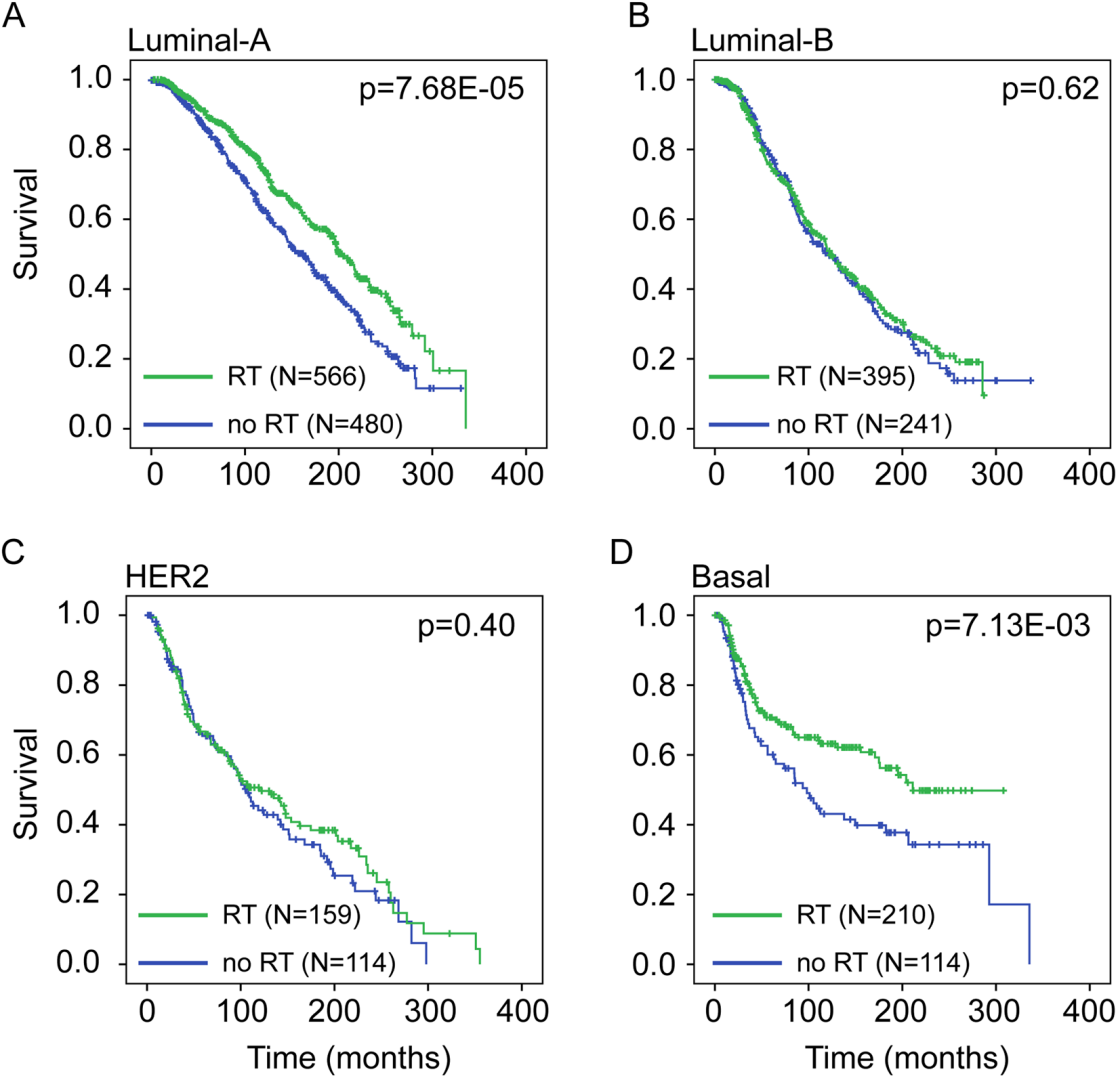
Interaction between molecular subtype and radiotherapy on overall survival in breast cancer patients by meta-analysis. Kaplan-Meier overall survival curves comparing survival for breast cancer patients who did and did not receive radiotherapy across different molecular subtypes: luminal-A (**A**), luminal-B (**B**), HER2 (**C**), basal (**D**). P-values were obtained using the log rank (Mantel-Cox) test.

BC is a heterogeneous disease and gene expression signatures have been developed that classify breast tumors into four relevant different molecular subtypes (luminal A, luminal B, HER2 and basal; see methods)^3–6^. Many studies have demonstrated an association between molecular subtype and patient prognosis^14,15^. The basal and HER2 subtypes are generally more aggressive and associated with poorer survival compared to normal-like and luminal breast tumors^16^. To investigate whether radiotherapy benefits patients equally among different molecular subtypes, we stratified our patient cohorts into different molecular subtypes based on the PAM50 molecular score^17^. Surprisingly, in the METABRIC cohort we found that radiotherapy increased patient survival only in the luminal A subtype (p = 3.66E-04), whereas a tendency for increased survival was observed for the basal subtypes (p = 0.13) (Figure S2). No survival benefit was observed for the other subtypes (luminal B: p = 0.78; HER2: p = 0.57) (Figure S2). In the TCGA cohort, radiotherapy significantly increased overall survival only in the basal subtype (p = 2.25E-04) (Figure S3). A tendency for survival benefit associated with radiotherapy was observed for the luminal A (p = 0.053) subtype (Figure S3). Again, no survival benefit was observed for the luminal B (p = 0.26) and HER2 (p = 0.32) subtypes (Figure S3). A meta-analysis combining the METABRIC and TCGA cohorts confirmed that radiotherapy significantly increased patient survival in the luminal A (p = 7.68E-05) and basal subtypes (p = 7.13E-03) (Fig. 2).

### Effect of age at diagnosis on patient survival after radiotherapy

**Figure 3:**
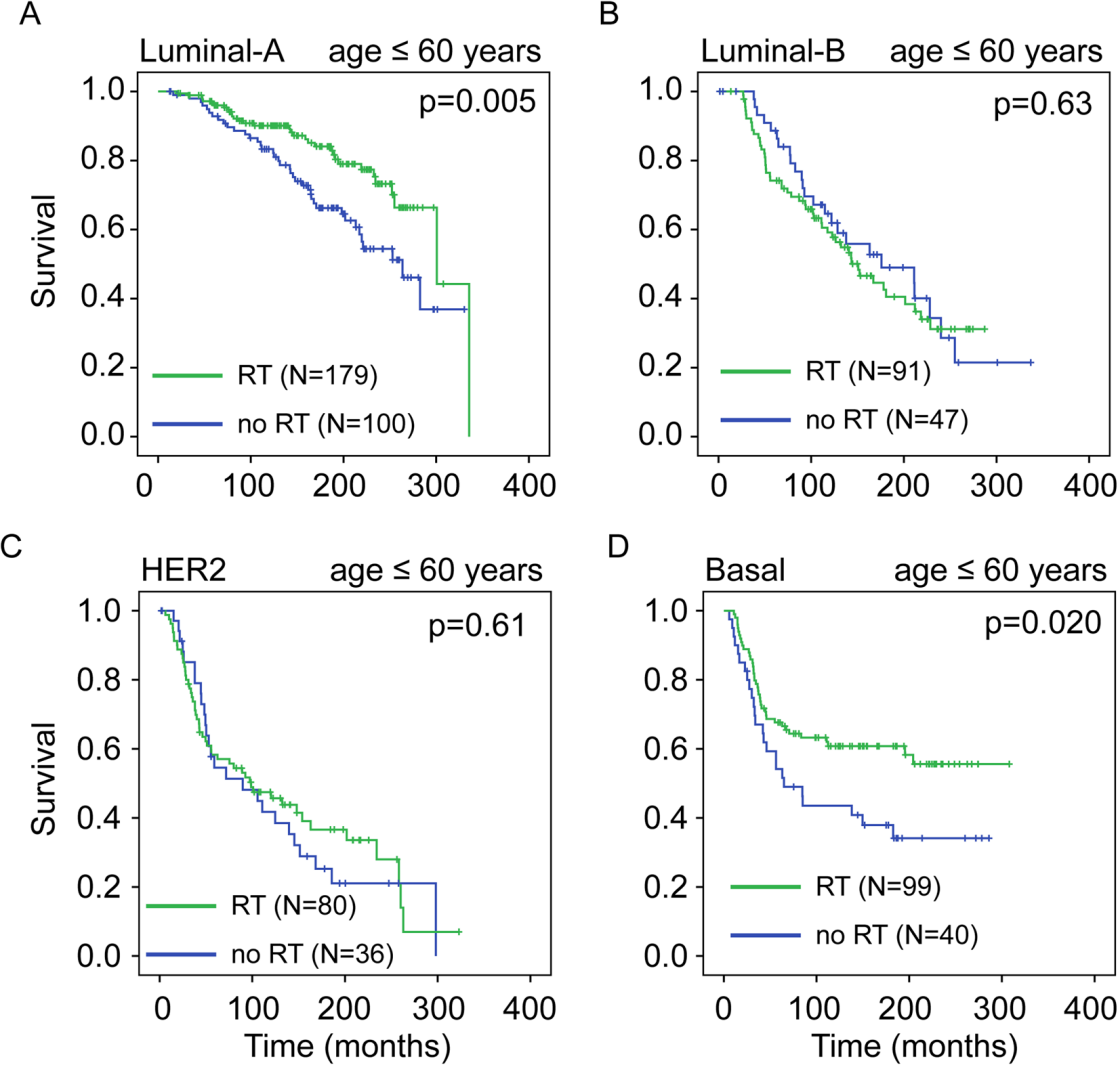
Effect of radiotherapy on overall survival in younger breast cancer patients across different molecular subtypes. Kaplan-Meier overall survival curves comparing survival for breast cancer patients diagnosed at age ≤60 years (“young”) who did and did not receive radiotherapy across different molecular subtypes: luminal-A (**A**), luminal-B (**B**), HER2 (**C**), and basal (**D**) (METABRIC cohort). P-values were obtained using the log rank (Mantel-Cox) test.

**Figure 4:**
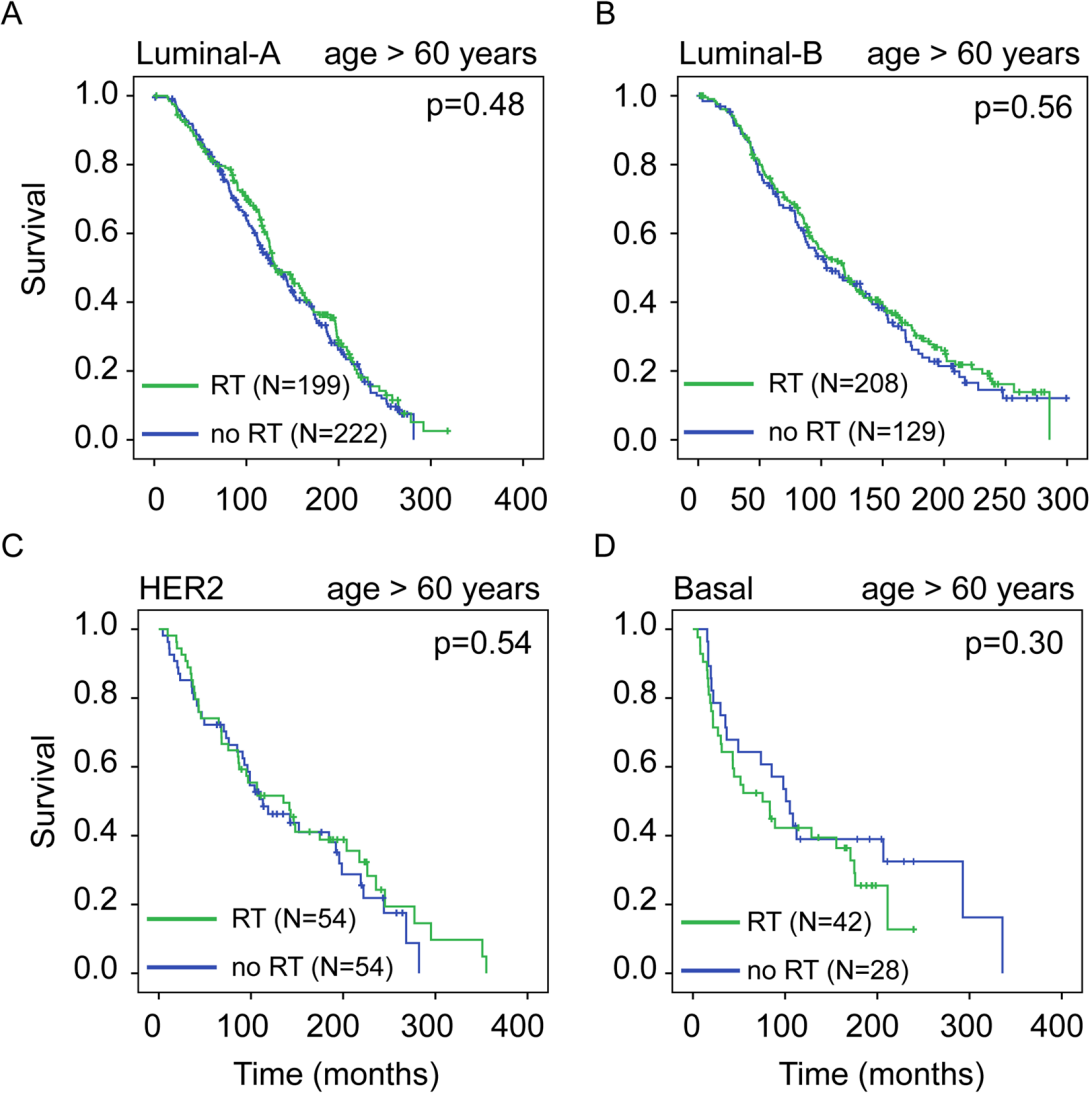
Effect of radiotherapy on overall survival in older breast cancer patients across different molecular subtypes. Kaplan-Meier overall survival curves comparing survival for breast cancer patients diagnosed at age > 60 years (“old”) who did or did not receive radiotherapy across different molecular subtypes: luminal-A (**A**), luminal-B (**B**), HER2 (**C**), basal (**D**) (METABRIC cohort). P-values were obtained using the log rank (Mantel-Cox) test.

Age at diagnosis is another well-known prognostic factor in BC^18,19^. We asked whether age at diagnosis in combination with molecular subtype could further clarify the survival benefit from radiotherapy. We split our patient cohorts into two age groups: “young” (age ≤60 years) and “old” (age >60 years). In the “young” group, radiotherapy significantly increased overall survival in luminal A (p = 0.005) and basal (p = 0.020) subtypes (Fig. 3). No survival benefit was observed in other subtypes (Fig. 3; luminal B: p = 0.63; HER2: p = 0.61). Surprisingly, in the “old” group, radiotherapy did not confer survival benefit for any molecular subtype (Fig. 4; luminal A: p = 0.48; luminal B: p = 0.56; HER2: p = 0.54, basal: p = 0.30).

## Discussion

In this study, we revisited the beneficial effects of radiotherapy on OS in BC patients by a population study using the METABRIC (N = 1980 patients) and TCGA (N = 1100 patients) databases. We first addressed the association between radiotherapy and overall survival time of breast cancer patients stratified by clinical stage, and found a significant increase in survival time in stage II breast cancer patients that received RT, but not in stage I and II-IV patients. Further stratification of patients by surgery type showed increased survival time in RT treated stage II breast cancer patients after lumpectomy compared to stage II patients after lumpectomy that did not receive RT treatment. RT only offered a marginal survival benefit in stage II breast cancer patients that underwent mastectomy. It has to be realized here that mastectomy patients do not routinely undergo radiation therapy, so this much smaller subgroup was probably selected for risk factors that we do not know and cannot analyze. Several studies have demonstrated improved survival and reduced local recurrence in patients receiving a combination of breast conserving therapy and RT compared to breast conserving therapy alone^20–23^. In addition, a number of studies have demonstrated in randomized controlled trials that breast conserving therapy in combination with RT is at least equivalent to mastectomy alone^24,25^. A retrospective cohort study including 5,335 women concluded that breast conserving surgery plus radiation resulted in better overall survival than mastectomy alone^26^. In addition, a Dutch population based study of 37,207 early breast cancer patients that showed that breast-conserving surgery plus radiotherapy showed improved survival compared to patients that underwent mastectomy^27^. However, the latter study could have been biased by “confounding by indication”.

We consistently found a significant survival benefit of radiotherapy for patients with luminal A or basal molecular subtypes who were diagnosed younger than 60 years of age. To translate our discoveries into clinical practice, future prospective clinical trials are warranted to validate our findings. Wang *et al*. found that adjuvant radiotherapy reduces the risk of relapse in breast tumors of the luminal A subtype, but not luminal B^28^. Consistent with this discovery, we also showed that tumors of the luminal A subtype showed a significant survival benefit after radiotherapy. However, such survival benefit was driven predominantly by younger patients (age at diagnosis ≤60 years), since no survival benefit was observed in the older age group (age at diagnosis >60 years). Regardless of age at diagnosis, patients with tumors of the luminal B subtype did not show any survival benefit from radiotherapy.

Similar to the luminal B subtype, patients with the HER2 subtype did not show survival benefit after radiotherapy. This data is consistent with previous observations^29^. In contrast, several groups did report an increased risk of recurrence for breast tumors overexpressing HER2 following radiotherapy^30,31^. This discrepancy could be due to additional heterogeneity within tumors of the HER2 subtype. Additional biomarkers are required to separate HER2 positive patients into radiosensitive and resistant cohorts.

Radiotherapy is a major treatment modality for patients with tumors of the basal molecular subtype, since their triple negativity offers no options for hormonal- or HER2 therapy. A few studies have shown increased mortality after radiotherapy in triple-negative breast tumors^29,30^. Surprisingly, we found a significant survival benefit associated with radiotherapy in patients with tumors of the basal subtype, especially those diagnoses in younger patients (age at diagnosis ≤60 years). Our result is consistent with a report that showed that adjuvant radiation was associated with improved overall survival in triple-negative breast cancer^32^. While not synonymous, basal-like breast cancers are predominantly triple-negative. Interestingly, in the present study improved survival was only observed in the lumpectomy group, not the mastectomy group.

The main limitations of our study are (1) incomplete clinical information in the TCGA dataset; for example, radiotherapy data was missing in ~20% of the data, (2) the clinical follow-up was different between METABRIC (approximately 30 years) and TCGA (majority less than 10 years), (3) confounding effects of systemic therapy were not taken into account in our study since this information was not present in the TCGA dataset and (4) our analysis is focused on OS due to incomplete clinical information for disease free survival. Nevertheless, from our studies we think we can conclude that there is a significant survival benefit of radiotherapy for especially younger patients with tumors of the luminal A and basal molecular subtypes, but not luminal B and HER2 driven subtypes. Future studies will need to be conducted to identify predictive biomarker panels that can accurately identify older, luminal A and basal subtype patients most likely to benefit from radiotherapy treatment, which can then be validated in prospective cohorts to ultimately improve clinical practice. Currently all breast cancer patients are treated with the same RT regimen with no prior knowledge as to which tumors are likely to respond and which are not. Our study takes an initial step towards more personalized treatment with the ultimate goal of reducing overtreatment with radiotherapy while retaining low breast cancer mortality.

## Methods

### Patient cohorts

Clinical data for TCGA and METABRIC cohorts were obtained from cBioPortal (http://www.cbioportal.org/data_sets.jsp) for 1100 breast cancer samples from The Cancer Genome Atlas consortium (TCGA)^33,34^ and for 1980 breast cancer samples from the Molecular Taxonomy of Breast Cancer International Consortium (METABRIC)^35,36^. TCGA specimens were collected from newly diagnosed patients with invasive breast adenocarcinoma at different US based tissue source sites. METABRIC primary fresh-frozen breast cancer specimens were obtained from tumor banks in the UK and Canada. Additional clinical data including PAM50, clinical stage, surgery, patient age at diagnosis and radiotherapy for the TCGA cohort was obtained from the UCSC Genome Browser and UCSC Cancer Browser (http://genome-cancer.ucsc.edu). Summary details of the two patient cohorts are presented in Table 1. With regard to PAM50 data, the small normal-like group was excluded because of doubt as to the relevance of this group (material analyzed may not have contained cancer) and the generally perceived lack of clinical relevance of this group. The claudin-low group was also excluded for being only available in METABRIC.

### Survival and statistical analysis

All survival and statistical analyses were performed using SPSS. Patients were stratified based on clinical stage, surgery type, age and molecular subtype (based on PAM50). Kaplan-Meier survival curves were generated to show differences in overall survival (p-values were generated using log rank (Mantel-Cox) test) between patients with and without radiotherapy. Multivariate analyses were carried out to examine whether radiotherapy has an independent benefit for survival when adjusting for other covariates (age, ER, PR, grade, tumor size) or the molecular subtypes using Cox proportional-hazard regression. Significance level was set at p < 0.05.

## Electronic supplementary material

Supplementary Figures

## References

[1] DeSantis C, Ma J, Bryan L, Jemal A (2014). Breast cancer statistics, 2013. CA: a cancer journal for clinicians.

[2] Siegel R, Ma J, Zou Z, Jemal A (2014). Cancer statistics, 2014. CA: a cancer journal for clinicians.

[3] Perou CM (2000). Molecular portraits of human breast tumours. Nature.

[4] Prat A, Parker JS, Fan C, Perou CM (2012). PAM50 assay and the three-gene model for identifying the major and clinically relevant molecular subtypes of breast cancer. Breast cancer research and treatment.

[5] Prat A (2010). Phenotypic and molecular characterization of the claudin-low intrinsic subtype of breast cancer. Breast cancer research: BCR.

[6] Prat A, Perou CM (2011). Deconstructing the molecular portraits of breast cancer. Molecular oncology.

[7] Holloway CM (2010). Technology as a force for improved diagnosis and treatment of breast disease. *Canadian journal of surgery*. Journal canadien de chirurgie.

[8] Duffy SW, Lynge E, Jonsson H, Ayyaz S, Olsen AH (2008). Complexities in the estimation of overdiagnosis in breast cancer screening. British journal of cancer.

[9] Glass AG, Lacey JV, Carreon JD, Hoover RN (2007). Breast cancer incidence, 1980-2006: combined roles of menopausal hormone therapy, screening mammography, and estrogen receptor status. Journal of the National Cancer Institute.

[10] Anampa J, Makower D, Sparano JA (2015). Progress in adjuvant chemotherapy for breast cancer: an overview. BMC medicine.

[11] Whelan TJ, Levine M, Julian J, Kirkbride P, Skingley P (2000). The effects of radiation therapy on quality of life of women with breast carcinoma: results of a randomized trial. Ontario Clinical Oncology Group. Cancer.

[12] Holli K, Saaristo R, Isola J, Joensuu H, Hakama M (2001). Lumpectomy with or without postoperative radiotherapy for breast cancer with favourable prognostic features: results of a randomized study. British journal of cancer.

[13] Lilla C (2007). Predictive factors for late normal tissue complications following radiotherapy for breast cancer. Breast cancer research and treatment.

[14] Reis-Filho JS, Pusztai L (2011). Gene expression profiling in breast cancer: classification, prognostication, and prediction. Lancet.

[15] Prat A (2015). Clinical implications of the intrinsic molecular subtypes of breast cancer. Breast.

[16] Sorlie T (2001). Gene expression patterns of breast carcinomas distinguish tumor subclasses with clinical implications. Proceedings of the National Academy of Sciences of the United States of America.

[17] Parker JS (2009). Supervised risk predictor of breast cancer based on intrinsic subtypes. Journal of clinical oncology: official journal of the American Society of Clinical Oncology.

[18] Nixon AJ (1994). Relationship of patient age to pathologic features of the tumor and prognosis for patients with stage I or II breast cancer. Journal of clinical oncology: official journal of the American Society of Clinical Oncology.

[19] Albain, K. S., Allred, D. C. & Clark, G. M. Breast cancer outcome and predictors of outcome: are there age differentials?*Journal of the National Cancer Institute*. *Monographs*, 35–42 (1994). 7999467

[20] Liljegren G (1994). Sector resection with or without postoperative radiotherapy for stage I breast cancer: five-year results of a randomized trial. Uppsala-Orebro Breast Cancer Study Group. Journal of the National Cancer Institute.

[21] Veronesi U (1993). Radiotherapy after breast-preserving surgery in women with localized cancer of the breast. The New England journal of medicine.

[22] Clark RM (1992). Randomized clinical trial to assess the effectiveness of breast irradiation following lumpectomy and axillary dissection for node-negative breast cancer. Journal of the National Cancer Institute.

[23] Fisher, B. & Redmond, C. Lumpectomy for breast cancer: an update of the NSABP experience. National Surgical Adjuvant Breast and Bowel Project. *Journal of the National Cancer Institute. Monograph*s, 7–13 (1992). 1627432

[24] Arriagada R, Le MG, Rochard F, Contesso G (1996). Conservative treatment versus mastectomy in early breast cancer: patterns of failure with 15 years of follow-up data. Institut Gustave-Roussy Breast Cancer Group. Journal of clinical oncology: official journal of the American Society of Clinical Oncology.

[25] Lichter AS (1992). Mastectomy versus breast-conserving therapy in the treatment of stage I and II carcinoma of the breast: a randomized trial at the National Cancer Institute. Journal of clinical oncology: official journal of the American Society of Clinical Oncology.

[26] Onitilo AA, Engel JM, Stankowski RV, Doi SA (2015). Survival Comparisons for Breast Conserving Surgery and Mastectomy Revisited: Community Experience and the Role of Radiation Therapy. Clinical medicine & research.

[27] van Maaren MC (2016). 10 year survival after breast-conserving surgery plus radiotherapy compared with mastectomy in early breast cancer in the Netherlands: a population-based study. The Lancet. Oncology.

[28] Wang Y (2011). A retrospective study of breast cancer subtypes: the risk of relapse and the relations with treatments. Breast cancer research and treatment.

[29] Kyndi M (2008). Estrogen receptor, progesterone receptor, HER-2, and response to postmastectomy radiotherapy in high-risk breast cancer: the Danish Breast Cancer Cooperative Group. Journal of clinical oncology: official journal of the American Society of Clinical Oncology.

[30] Nguyen PL (2008). Breast cancer subtype approximated by estrogen receptor, progesterone receptor, and HER-2 is associated with local and distant recurrence after breast-conserving therapy. Journal of clinical oncology: official journal of the American Society of Clinical Oncology.

[31] Stal O (1995). c-erbB-2 expression and benefit from adjuvant chemotherapy and radiotherapy of breast cancer. European journal of cancer.

[32] Steward LT, Gao F, Taylor MA, Margenthaler JA (2014). Impact of radiation therapy on survival in patients with triple-negative breast cancer. Oncology letters.

[33] Cerami E (2012). The cBio cancer genomics portal: an open platform for exploring multidimensional cancer genomics data. Cancer discovery.

[34] Gao J (2013). Integrative analysis of complex cancer genomics and clinical profiles using the cBioPortal. Science signaling.

[35] Curtis C (2012). The genomic and transcriptomic architecture of 2,000 breast tumours reveals novel subgroups. Nature.

[36] Pereira B (2016). The somatic mutation profiles of 2,433 breast cancers refines their genomic and transcriptomic landscapes. Nature communications.

