# Supplementary material for: Revisiting the impact of age and molecular subtype on overall survival after radiotherapy in breast cancer patients
Source: Sci Rep. 2017 Oct 3;7:12587. doi: 10.1038/s41598-017-12949-5 (PMC5626767; doi:10.1038/s41598-017-12949-5)
Supplement: Supplementary file 1 — Supplementary Figures [file 41598_2017_12949_MOESM1_ESM.pdf]

Revisiting the impact of age and molecular subtype on overall survival after radiotherapy in breast cancer patients

Jian-Hua Mao, Paul J. van Diest, Jesus Perez-Losada and Antoine M. Snijders

Supplementary Figures S1-S3

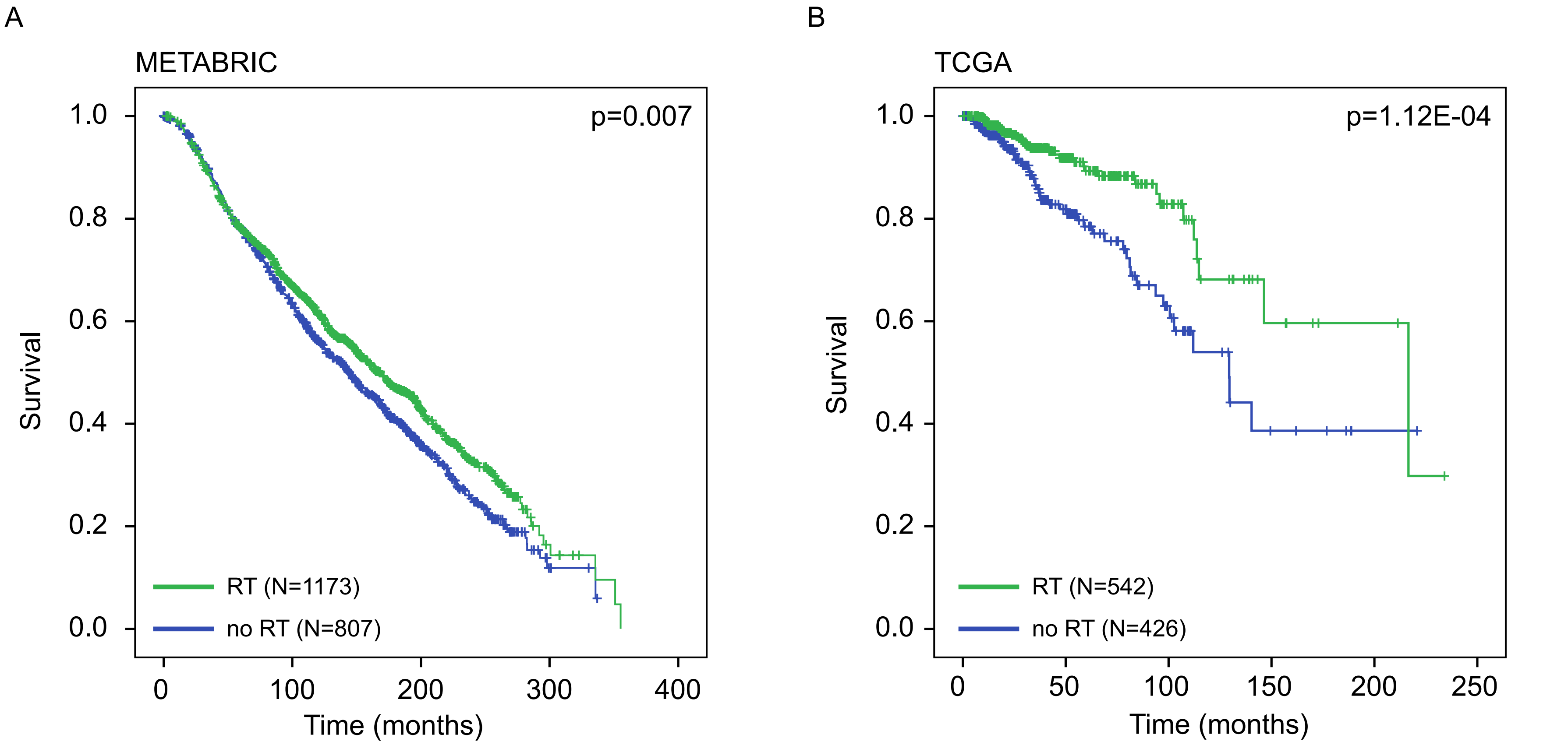

Figure S1. Effect of radiotherapy on overall survival in breast cancer patients.
A. Kaplan-Meier overall survival curve of 1980 breast cancer patients (METABRIC) with (N=1173 patients) and without (N=807 patients) radiotherapy (p=0.007). B. Kaplan-Meier overall survival curve of 968 breast cancer patients (TCGA) with (N=542 patients) and without (N=426 patients) radiotherapy (p=1.12E-04). P-values were obtained using the log rank (Mantel-Cox) test.

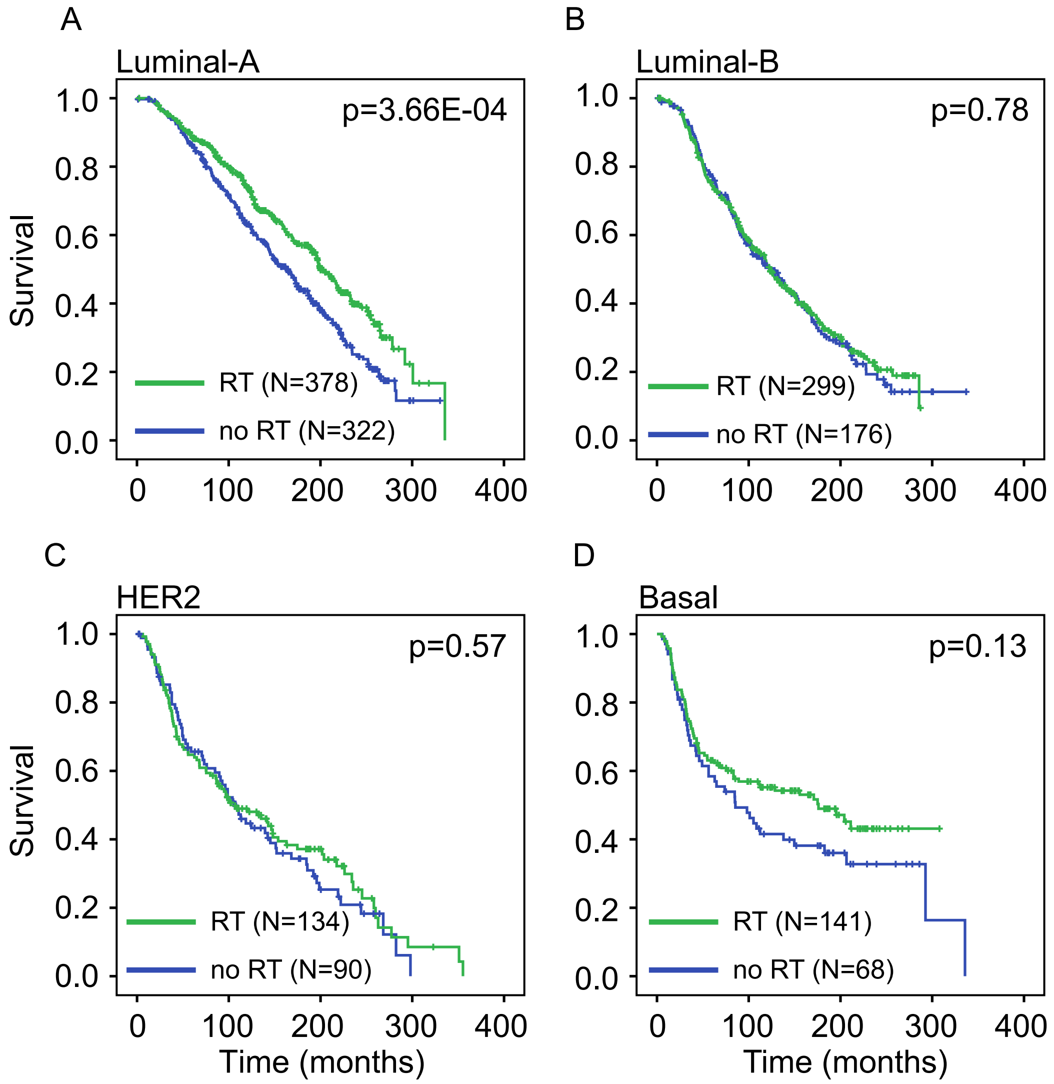

Figure S2. Interaction between molecular subtype and radiotherapy on overall survival in breast cancer patients from the METABRIC cohort. Kaplan-Meier overall survival curves comparing survival for breast cancer patients who did and did not receive radiotherapy across different molecular subtypes: luminal-A (A), luminal-B (B), HER2 (C), basal (D). P-values were obtained using the log rank (Mantel-Cox) test.

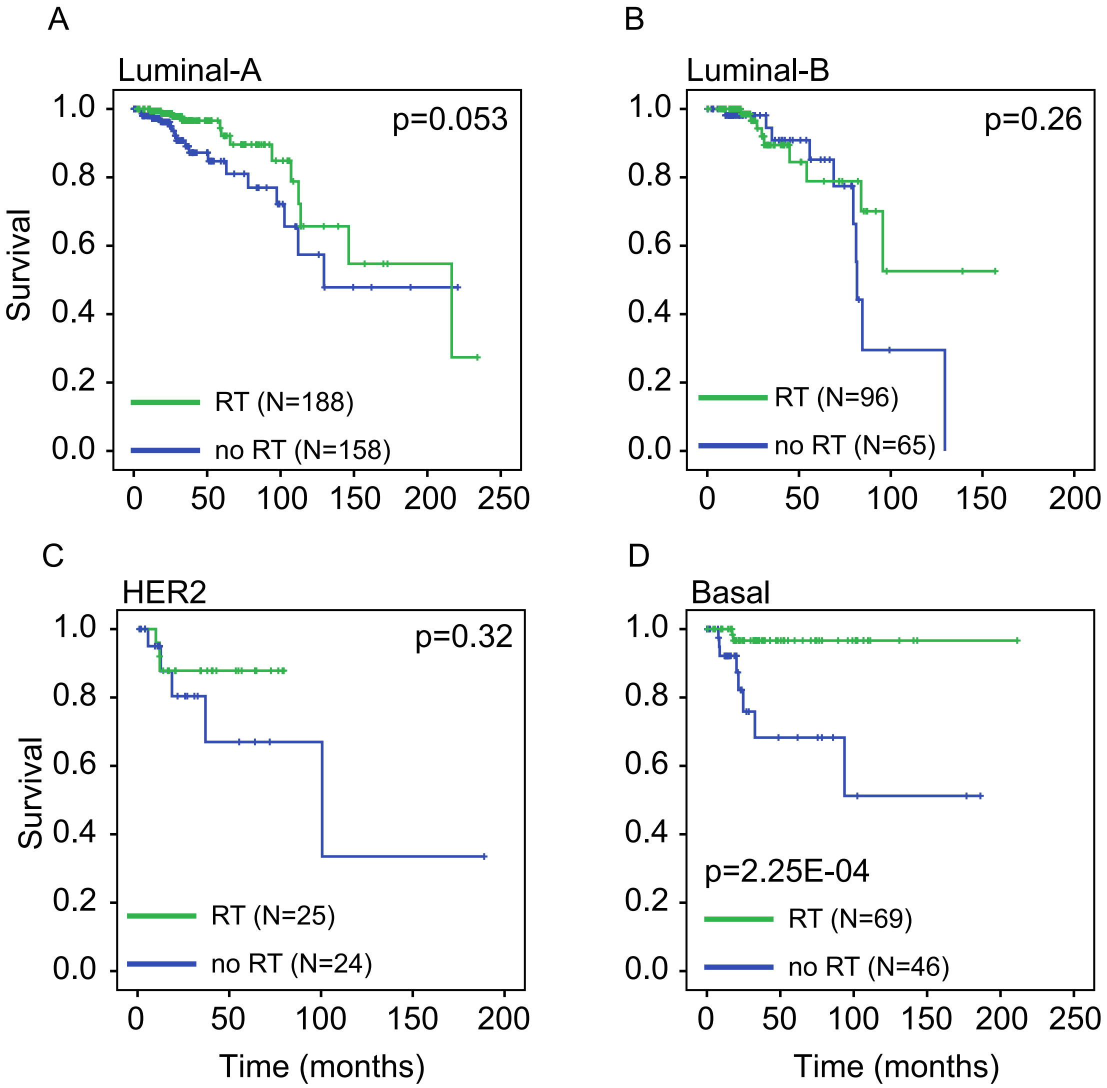

Figure S3. Interaction between molecular subtype and radiotherapy on overall survival in breast cancer patients from the TCGA cohort. Kaplan-Meier overall survival curves comparing survival for breast cancer patients who did and did not receive radiotherapy across different molecular subtypes: luminal-A (A), luminal-B (B), HER2 (C) and basal (D). P-values were obtained using the log rank (Mantel-Cox) test.
